# Living Alone and Homelessness as Predictors of 30-Day Potentially Preventable Hospital Readmission

**DOI:** 10.5888/pcd16.180189

**Published:** 2019-02-07

**Authors:** Emiline LaWall, Yan Yan Wu, Victoria Y. Fan, Melinda Ashton, Tetine Sentell

**Affiliations:** 1 Hawai‘i Pacific Health, Honolulu, Hawai‘i; 2 University of Hawai‘i at Mānoa, Honolulu, Hawai‘i

## Abstract

**Introduction:**

The effect of social factors on health care outcomes is widely recognized. Health care systems are encouraged to add social and behavioral measures to electronic health records (EHRs), but limited research demonstrates how to leverage this information. We assessed 2 social factors collected from EHRs — social isolation and homelessness — in predicting 30-day potentially preventable readmissions (PPRs) to hospital.

**Methods:**

EHR data were collected from May 2015 through April 2017 from inpatients at 2 urban hospitals on O‘ahu, Hawai‘i (N = 21,274). We performed multivariable logistic regression models predicting 30-day PPR by living alone versus living with others and by documented homelessness versus no documented homelessness, controlling for relevant factors, including age group, race/ethnicity, sex, and comorbid conditions.

**Results:**

Among the 21,274 index hospitalizations, 16.5% (3,504) were people living alone and 11.2% (2,385) were homeless; 4.2% (899) hospitalizations had a 30-day PPR. In bivariate analysis, living alone did not significantly affect likelihood of a 30-day PPR (16.6% [3,376 hospitalizations] without PPR vs 14.4% [128 hospitalizations] with PPR; *P* = .09*).* However, documented homelessness did show a significant effect on the likelihood of 30-day PPR in the bivariate analysis (11.1% [2,259 hospitalizations] without PPR vs 14.1% [126 hospitalizations] with PPR; *P* = .006). In multivariable models, neither living alone nor homelessness was significantly associated with PPR. Factors that were significantly associated with PPR were comorbid conditions, discharge disposition, and use of an assistive device.

**Conclusion:**

Homelessness predicted PPR in descriptive analyses. Neither living alone nor homelessness predicted PPR once other factors were controlled. Instead, indicators of physical frailty (ie, use of an assistive device) and medical complexity (eg, hospitalizations that required assistive care post-discharge, people with a high number of comorbid conditions) were significant. Future research should focus on refining, collecting, and applying social factor data obtained through acute care EHRs.

**Summary**

**What is already known about this topic?**

Health systems are encouraged to add social and behavioral measures to electronic health records (EHRs), but there is limited research that demonstrates how to leverage this information.

**What is added by this report?**

We assessed 2 social factors collected from EHRs — social isolation and homelessness — in predicting 30-day potentially preventable readmissions to hospital.

**What are the implications for public health practice?**

Because social and behavioral factors affect patient health, health care systems must rethink the way these measures are defined and captured in EHRs. Our study illustrates how social factors (ie, homelessness and social isolation) can be leveraged for predictive modeling of acute care outcomes.

## Introduction

Because of widespread recognition of the relationship between social factors and health care outcomes, the Institute of Medicine (IOM) provided recommendations for social and behavioral domain measures to be documented in the electronic health record (EHR) (1). Many health systems now collect some data on social factors, but practical and logistical questions remain, including how to feasibly and systematically collect such data during routine clinical care and how to use these data for more effective population health management (1–3).

“Social connection and isolation” was one domain recommended by IOM (4). A lack of social relationships has been associated with numerous health outcomes, including illness, functional decline, and death (5–7). Two substantial gaps exist in the literature. First, limited research has been done on whether social isolation plays a role in overall health care use (8,9). Second, the measure of social isolation is typically collected outside the EHR, via self-reported survey or interview (5,10–12). Thus, these measures are rarely found in analyses of administrative inpatient data.

Another critical social factor potentially contributing to inpatient health care use and readmission is homelessness. Homeless status also is not routinely collected by health care systems in the EHR, and it has been associated in several recent studies with readmissions and with overall illness and early death (13,14).

Our objective was to examine whether variables for social isolation and homelessness as captured in a health system’s EHRs over 2 years predicted 30-day potentially preventable readmission (PPR), a key measure of health care quality (15). We hypothesized that people who were documented as living alone or homeless would have a higher likelihood of PPR than those without these designations.

## Methods

### Data and inclusion criteria

We collected EHR data for 25,717 people aged 18 or older who had at least 1 inpatient hospitalization from May 1, 2015, through April 30, 2017, at 2 midsized urban hospitals on O‘ahu, Hawai‘i . Any hospitalizations that resulted in death were excluded from the analysis (n = 1,101). These data uniquely identified individuals longitudinally. By using this unique identifier, each person’s first inpatient hospitalization during the study period was identified. These initial hospitalizations were flagged to indicate whether they resulted in a 30-day PPR (yes/no) by using the 3M PPR methodology (16). The 3M PPR methodology has been extensively used and validated (17). People with subsequent readmission encounters were excluded from the sample (n = 949) to prevent high-use patients from biasing our understanding of demographic and clinical factors predicting PPR in general. The institutional review board of Hawai‘i Pacific Health approved our study.

**Figure 1:**
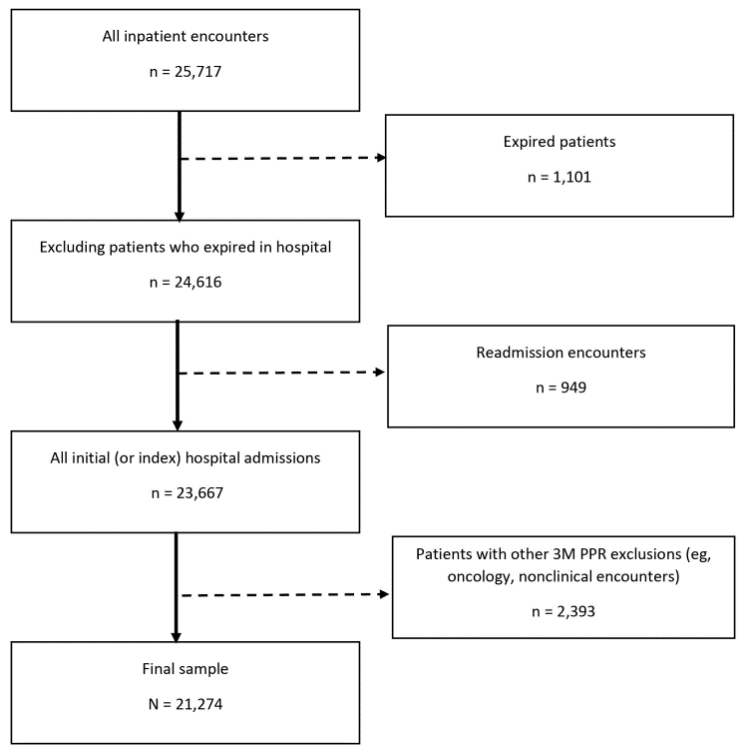
Selection criteria for the predictive model for all inpatient encounters (N = 25,717) collected from 2 urban hospitals in Hawai’i. Abbreviation: PPR, potentially preventable readmission.

Hospitalizations that were not considered eligible under the 3M PPR methodology were also excluded (n = 2,393). These were, for example, people re-admitted for conditions that were not clinically related (eg, a hospital admission for pneumonia followed by a hospitalization for an appendectomy), people admitted with multiple traumas (where multiple hospitalization may be medically necessary), and people currently in chemotherapy treatment and likely to return to the hospital (18). For the final analysis, a total of 21,274 inpatient hospitalizations were used (Figure 1). SAS Version 9.4 (SAS Institute) was used to complete both descriptive statistics and multivariable modeling. Our outcome was a binary variable that indicated whether a person’s initial hospitalization during the study period resulted in a 30-day PPR (yes/no) according to 3M PPR methods (18,19).

**Independent variables.** At both hospitals included in our study, the “lives alone” identifier has been collected consistently since October 2015. Nurses ask patients to identify who they live with as a part of the admission process. Case managers are required to populate this field if it is missed during the admission process. Nurses and case managers are allowed to select 1 or more of the following options: alone, caregiver, family, parent, partner, roommate, spouse, or other. We used the information from this field to categorize patients into 2 groups, either as people who live alone or people who live with others.

For the independent variable of homelessness, the reporting team created a wild card search for “*homeless*” in numerous free-text fields for “residence” and collated them as a 0/1 flag. The information from this field was used to categorize patients into 2 groups, either as people with documented homelessness or people without documented homelessness.

**Control variables.** Several demographic factors were considered for control variables, including race/ethnicity (white, Chinese, Japanese, Filipino, Native Hawaiian, other Pacific Islander, and other), age group (<65 y and ≥65 y), sex (male/female), and insurance (public [Medicare or Medicaid], private, or other). Several clinical factors were also collected, including admission source (eg, whether a person came to the hospital through the emergency department, from another health care facility, or via physician referral), length of hospitalization, length of stay in intensive care unit, case mix index (a severity of illness weight assigned by Centers for Medicare & Medicaid Services on the basis of a patient’s principal diagnosis) whether patients had a surgical encounter (yes/no), whether patients have a device to assist with mobility (yes/no), the Elixhauser comorbidity score (a method of categorizing co-occurring diseases or disorders in addition to the primary diagnosis) (20), presence of mental health diagnoses (yes/no), and discharge disposition (eg, discharged to home, hospice, or skilled nursing facility). Data for all independent and control variables were taken from the index admission.

### Statistical analysis

For the first stage of analysis, we summarized data on patients with and without 30-day PPR with descriptive statistics by using χ^2^ tests or Fisher exact tests (for categorical variables) and 2-sample *t* tests (for continuous variables). Two-tailed tests using an α of .05 were used to assess the significance for these analyses in predicting 30-day PPR; significant factors were included as control variables in the logistic regression equations.

For the second stage of analysis, a logistic regression model was developed to estimate the likelihood of PPR after the index hospital admission by using control variables identified through the descriptive statistics as being significant (*P* < .05); we also used the 2 independent variables of interest, lives alone (yes/no) and homeless (yes/no). We also tested for an interaction between our 2 focal independent variables: lives alone and homeless. The interaction was not significant (*P* = .99) and was not included in the final multivariable model.

## Results

Among the 21,274 index hospitalizations, 16.5% (3,504) were people living alone and 11.2% (2,385) were homeless. Of the index hospitalizations, 4.2% (899) of hospitalizations had a 30-day PPR. Of the index hospitalizations, 21,251 had documentation around whether patients lived alone (documentation for living alone was not populated for 23 cases; these encounters were excluded from bivariate analysis). Of the 21,251 that had documentation around living alone, 20,357 encounters did not have a PPR, and 894 encounters did. In bivariate analysis, living alone did not show significance for PPR. People who lived alone represented 16.6% of non-PPR encounters (3,376 of 20,357) and 14.4% of PPR encounters (128 of 894, *P* = .09).

Documentation about whether the patient was homeless was available for 21,267 of the 21,274 index hospitalizations (documentation for homelessness was not populated for 7 cases, and these encounters were excluded from bivariate analysis). Of the 21,267 that had documentation around homelessness, 20,370 encounters did not have a PPR, and 897 encounters did. In the descriptive analyses, people who were documented as homeless were significantly more likely to have a PPR encounter. People who were documented as homeless represented 11.1% of non-PPR encounters (2,259 of 20,370) and 14.1% of PPR encounters (126 of 897, *P* = .006). We found no significant differences for PPR in age, sex, length of stay, length of stay in intensive care unit, case mix index, or the presence of a mental health diagnosis (Table 1).

**Table 1:** Characteristics of Patients With and Without a 30-day Potentially Preventable Hospital Readmission (PPR) (N = 21,274) Following an Index Hospitalization, Two Urban Hospitals, Hawai‘i , May 2015–April 2017^a^

Variable	All Patients (n = 21,274)		
	No PPR	Have PPR	*P* Value
**Lives alone**			
Yes	3,376 (16.6)	128 (14.4)	.09
No	16,981 (83.4)	766 (85.6)	
**Homeless**			
Yes	2,259 (11.1)	126 (14.1)	.006
No	18,111 (88.9)	771 (86.0)	
**Age, y**			
<65	9,312 (45.7)	394 (43.8)	.27
≥65	11,063 (54.3)	505 (56.1)	
**Sex**			
Male	10,611 (52.1)	480 (53.4)	.44
Female	9,762 (47.9)	419 (46.6)	
**Race/ethnicity**			
White	5,133 (21.2)	200 (22.3)	.02
Chinese	917 (4.5)	43 (4.8)	
Filipino	3,316 (16.3)	143 (15.9)	
Hawaiian	2,737 (13.4)	160 (17.8)	
Japanese	5,204 (25.5)	214 (23.8)	
Other Pacific Islander	1,353 (6.6)	65 (7.2)	
Other	1,715 (8.4)	74 (8.23)	
**Insurance**			
Private	6,902 (33.9)	266 (29.6)	<.001
Public	13,241 (65.0)	618 (68.9)	
Other	232 (1.14)	14 (1.6)	
**Admission source**			
Emergency	15,097 (74.1)	721 (80.2)	<.001
Referral	3,743 (19.4)	110 (12.2)	
Transfer	1,521 (7.5)	67 (7.4)	
**Length of stay**	5.5–6.6	7.0 ± 6.7	.99
**ICU days^b^**	0.4–1.7	0.5 ± 1.8	.99
**Case mix index^c^**	1.9–1.4	1.9 ± 1.4	.99
**Surgery**			
Yes	7,255 (35.6)	275 (30.6)	.002
No	13,120 (64.4)	624 (69.4)	
**Uses assistive device^d^**			
Yes	11,453 (56.3)	372 (41.5)	<.001
No	8,876 (43.7)	524 (58.5)	
**Elixhauser comorbidity score (number of co-occurring disorders in addition to primary diagnosis)**			
0	848 (4.2)	7 (0.8)	<.001
1–3	6,668 (32.7)	156 (17.4)	
4–6	6,206 (30.5)	234 (26.1)	
7–9	3,850 (18.9)	225 (25.1)	
≥10	2,805 (13.8)	275 (30.7)	
**Mental health**			
Yes	14,797 (72.6)	641 (71.3)	.38
No	5,578 (27.4)	258 (29.70)	
**Discharged to**			
Home/self-care	14,891 (73.1)	650 (72.3)	<.001
Hospice	498 (2.4)	1 (0.1)	
Skilled nursing facility	2,347 (11.5)	94 (10.5)	
Other facility	2,639 (13.0)	154 (17.1)	

a Values are number (percentage) unless otherwise indicated. Where null values existed, index hospitalizations were removed from binary analysis. Null values for each are lives alone (n = 23), homelessness (n = 7), sex (n = 2), insurance (n = 1), admission (n = 15), and assistive device (n = 49).

b ICU days indicates number of days spent in an intensive care unit.

c The severity of illness weight assigned by Centers for Medicare & Medicaid Services on the basis of a patient’s principle diagnoses.

d Requires a device to aid with mobility (eg, wheelchair, cane, walker).

Thirty-day PPR differed across racial/ethnic groups (*P* = .02); people of documented Native Hawaiian race had proportionally higher rates of 30-day PPR compared with other racial/ethnic groups. A significant difference was also seen in 30-day PPR by insurance type (*P* < .001). Those with public insurance were more likely to have a 30-day PPR than those with private insurance. Other significant variables in descriptive statistics were having a surgical encounter at index hospitalization (*P* = .002), having a device to assist with mobility at index admission (*P* < .001), admission through the emergency department versus physician referral or transfer (*P* < .001), discharge to a skilled nursing facility or long-term care facility at index admission versus discharged home to self-care (*P* < .001), and Elixhauser comorbidity score (*P* < .001).

**Multivariable model**. In the multivariable model predicting 30-day PPR (Table 2), neither the variable for lives alone (OR = 1.17; 95% CI, 0.96–1.42) nor homelessness (OR = 0.87; 95% CI, 0.71–1.07) was a significant predictor of 30-day PPR. Factors significantly associated with 30-day PPR were index admission source (those with physician referral were less likely to have a 30-day PPR than those admitted through the emergency department [OR = 0.73; 95% CI, 0.58–0.92]), use of a device for mobility-assistance at index admission (those with a device were less likely to have a 30-day PPR than those who did not have a device [OR = 0.72; 95% CI, 0.62–0.84]), Elixhauser comorbidity score at index admission (those with ≥10 comorbidities were more likely to have a 30-day PPR than those who did not have comorbidities [OR = 9.30; 95% CI, 4.30–20.00]), and discharge disposition at index admission (those admitted to a skilled nursing facility were less likely than those who were discharged to home or self-care to have a 30-day PPR [OR = 0.65; 95% CI, 0.51–0.82]).

**Table 2:** Multivariable Logistical Model Predicting Having a 30-Day Potentially Preventable Hospital Readmission Following an Index Hospitalization (N = 21,124), Two Urban Hospitals, Hawai‘i, May 2015–April 2017^a^

Variable	Odds Ratio (95% Confidence Interval)
**Lives alone**	
Yes	1.17 (0.96–1.42
No	1 [Reference]
**Homeless**	
Yes	0.87 (0.71–1.07)
No	1 [Reference]
**Admission source**	
Emergency	1 [Reference]
Referral	0.73 (0.58–0.92)
Transfer	1.04 (0.80–1.07)
**Age, y**	
<65	1.13 (0.96–1.32)
≥65	1 [Reference]
**Sex**	
Male	0.93 (0.81–1.07)
Female	1 [Reference]
**Race/ethnicity**	
Caucasian	1 [Reference]
Chinese	1.14 (0.81–1.60)
Filipino	0.95 (0.76–1.18)
Hawaiian	1.12 (0.90–1.40)
Japanese	1.01 (0.82–1.23)
Other Pacific Islander	0.94 (0.80–1.11)
Other	1.12 (0.85–1.48)
**Insurance**	
Private	1 [Reference]
Public	1.29 (1.09–1.53)
Other	0.61 (0.22–1.66)
**Admission source**	
Emergency	1 [Reference]
Referral	0.73 (0.58–0.92)
Transfer	1.04 (0.80–1.07)
**Surgery**	
Yes	0.94 (0.80–1.11)
No	1 [Reference]
**Uses assistive device^b^**	
Yes	0.72 (0.62–0.84)
No	1 [Reference]
**Elixhauser comorbidity score (number of co-occurring disorders in addition to primary diagnosis)**	
0	1 [Reference]
1–3	2.77 (1.24–5.72)
4–6	3.98 (1.86–8.51)
7–9	5.91 (2.75–12.70)
≥10	9.30 (4.30–20.00)
**Discharged to**	
Home/self-care	1 [Reference]
Hospice	0.03 (0–0.21)
Skilled nursing facility	0.65 (0.51–0.82)
Other facility	1.10 (0.91–1.33)

a Excludes 150 patients with missing response or explanatory variables.

b Requires a device to aid with mobility (eg, wheelchair, cane, walker).

**Figure 2:**
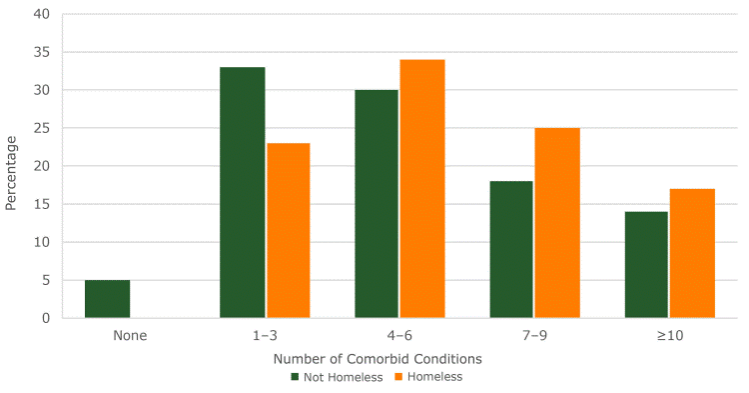
Number and distribution by percentage of Elixhauser comorbidity counts (20) for nonhomeless and homeless patients for 2 urban hospitals in Hawai‘i . Comorbidities are the existence of multiple chronic conditions in addition to the principal diagnosis or reason for hospitalization.

In our study, the percentage of homeless people with comorbidities was significantly higher than it was in those who were not homeless (*P* < .001) (Figure 2). Patients who were homeless had a higher percentage of 4 or more comorbidities (76%) than their counterparts who were not homeless (62%). None of the homeless patients studied had zero comorbidities.

**Table:** 

No. Comorbidities	Not Homeless, %	Homeless, %
0	5	0
1–3	33	23
4–6	30	34
7–9	18	25
≥10	14	17

## Discussion

Despite overwhelming evidence that social factors are critical determinants of health, these factors are rarely captured routinely in health system EHRs (21). Several major entities, including the Institute of Medicine and the Centers for Medicare & Medicaid Services, are calling for clinical and public health practitioners to examine ways to effectively collect data in these social and behavioral domains, including in acute care settings (4). Both the methods of collecting these data and the findings from this research add useful new evidence to the growing literature about how to effectively capture these social factors as a part of routine clinical care.

Living alone and homelessness were relatively common in our inpatient sample. Almost 20% of the sample lived alone, and over 10% were documented as homeless. Homelessness predicted PPR in descriptive analyses, although neither of these social factors predicted PPR once other factors were controlled. Instead, indicators of physical frailty (having a device to assist with mobility at index admission) and greater illness were significant; this finding is congruent with a vast body of evidence that identified the importance of comorbidity in predicting PPR, even to the exclusion of other social factors that commonly predict health disparities, particularly race/ethnicity (19,20).

Both the social domains of living alone (isolation) and homelessness may challenge the practicality of data collection. Social isolation is complex and difficult to define (22). Comprehensively measuring social relationships requires “consideration of both structural (eg, marital status, living arrangements) and functional (eg, emotional, perceived) aspects” (22) of isolation or the quantity and quality of relationships (7).

In previous literature, social isolation was identified as a contributor to all-cause mortality and higher health care use, particularly in adults aged over 65 (5,23). However, many of these studies were based on patient interview or self-reported surveys (9). Our study contributes new information by examining social isolation from administrative data documented by clinicians. Of course, a trade-off of using an administrative data point is that it may not capture the same level of complexity and nuance that can be garnered from patient interview or self-reported survey; this may explain our study findings. Further research is needed to understand how to best implement the complex measure of social isolation and other social determinants of health in a manner that is practical and useful for clinical care but nuanced enough to capture data in relevant domains.

In the Hawai‘i health care system used in our study, front-line clinicians consistently documented and referenced the measure of whether patients lived alone to determine whether additional post-discharge care would be required (eg, identified caregivers, made frequent post-discharge telephone calls). Our study helped to determine whether this simple proxy measure predicted 30-day PPR. For the 2 hospital facilities in our study, living alone did not predict 30-day PPR, which could indicate that people living alone may have some protective factors. For example, living alone may be an indicator of a person’s level of independence.

Another factor to consider when interpreting these data is how the front-line staff uses the information to inform a patient’s treatment plan. At the 2 hospitals in our study, case managers assessed each patient within 24 hours of admission. Then, depending on reason for admission — comorbidities, functional changes, medical prognosis, support system — and insurance coverage, patients were referred to a medical social worker for access to community resources or long-term care planning. According to email messages from Judy Suzuki of Straub Medical Center and Rochelle Day of Pali Momi Medical Center (July 2018), shelter options were offered to homeless patients, and if they refused, refusal was documented. An interesting area of future study would be how referral resources contribute to 30-day PPR and to 30-day PPR trends for people who were offered these services but refused the referral.

Several regionally relevant factors may also explain why social isolation was not a significant predictor of 30-day PPR. First, the state of Hawai‘i has the highest national percentage of multigenerational households, 11.1% of all family households (26). This high percentage could be due to cultural or economic reasons (eg, high cost of living) (26). Accordingly, our data set may have a smaller percentage of people living alone than other areas of the United States. Living alone in the state of Hawai‘i may thus indicate certain protective factors, such as higher income.

A second factor unique to our data set is a discharge-planning bill (27) that the Hawai‘i state legislature passed in March 2016 (during our data collection). This bill requires hospitals to adopt and maintain discharge policies consistent with federal regulations and asks providers to identify and document a designated caregiver for each patient before discharge. Identification of a designated caregiver possibly improved readmission outcomes, particularly among people who live alone. Although data are not yet available on how this legislation has affected Hawai‘i ’s hospital readmission rates, similar legislation was introduced in other states and yielded a 25% decrease in 90-day readmission rates compared with usual care (28).

Although homelessness was a significant predictor of readmissions in crude analyses, it was not significant once other control variables were added. This outcome ran counter to our initial hypothesis. In a previous study (29), homeless people were found to be 3 times more likely to be readmitted than their peers who were matched for age, sex, and clinical acuity (29). Homelessness was found to increase both emergency department and inpatient visits (30). People experiencing homelessness are expected to have higher readmissions for various reasons, including higher comorbidity rates associated with inadequate living conditions and limited access to primary care (13). In our study, the percentage of homeless people with 4 or more comorbidities was significantly higher than the percentage of people who were not homeless. The strength of the relationship between comorbidity and readmissions possibly masked the effect of homelessness in the multivariate models. Future research could use mediation analyses to better disentangle the causal relationship between homelessness, comorbidities, and readmissions.

For the variable of homelessness, a wild card search for the word *homeless* was created in free-text fields related to residence. This method of documenting homelessness probably significantly underestimates homelessness, and it introduces classical measurement error leading to attenuation bias, making it harder to detect an effect. For example, the method does not capture data on people who provide a homeless shelter for their residential address. Similarly, it does not address those with unstable housing situations (such as sleeping on a friend’s couch), who may also be vulnerable to readmission.

Our study had limitations. Our data were limited to the frequency of 30-day PPR in 1 hospital system in Hawai‘i , which may limit generalizability. It may also underreport the true frequency of 30-day PPR per patient, particularly for people with subsequent hospital encounters at other health care systems. Future research using other measures of readmission may reveal distinct patterns by readmission type. Other potential confounding variables for which we lacked data were preferred language, compliance to post-discharge medication, and income. People with a low income could lack resources to pay for housing, leading to homelessness; low income could be indirectly associated with having the necessary resources to pay for preventive services, which in turn leads to hospital readmissions. Thus, the effect of homelessness on readmissions in this analyses may be larger in magnitude than in reality, given that we did not control for income.

Preventable readmissions are an important policy focus for the Centers for Medicare & Medicaid Services, and readmissions can be measured in various ways. In our study, we selected the 3M PPR methodology as the outcome variable of interest because it offers the logic to examine potentially preventable readmissions (ie, those clinically related to a prior admission) rather than 2 clinically unrelated, but merited, encounters (eg, appendicitis, hip fracture). Preventability is particularly useful from the perspective of a health care facility because it identifies targets to improve quality of care and reduce readmissions. Future studies should consider additional readmission metrics.

Given that our independent variable of “lives alone” did not appear to have predictive value for all-cause 30-day PPR, modifying the granularity of social isolation measures could be a fruitful area of future study. Although identification of people living alone can predict both illness and death, it does not provide the same granularity of whether the patient perceives loneliness in addition to living alone (9,22,23). Further research is needed to understand how to best operationalize more robust social measures, including social isolation, in the acute care setting.

Our study has similar limitations with respect to capturing data on homelessness in the EHR. Free-text capture of homelessness underreports those who are unstably housed or those who are currently living in shelters. The literature notes limitations and mixed approaches currently used to capture data on homelessness in the EHR, ranging from using a listing of a shelter address to the number of home address changes as a proxy for being unstably housed (14,29,30). One study by Doran and colleagues suggests that homelessness screening should be completed by multiple practitioners to ensure redundancy and increase rate of data capture (14). Identifying and implementing standard approaches to capturing data on homelessness in the EHR is critical for health policy and programming.

Because social and behavioral factors affect patient health, health care systems must rethink the way these measures are defined and captured in EHRs (21). Our study illustrates how social factors (ie, homelessness and social isolation) can be leveraged for predictive modeling of acute care outcomes. Further research is needed to refine and operationalize social and behavioral domains in a way that can be practically collected in care, specifically for acute care populations.
